# The Short-Term Effects of Soybean Intake on Oxidative and Carbonyl Stress in Men and Women

**DOI:** 10.3390/molecules18055190

**Published:** 2013-05-07

**Authors:** Peter Celec, Július Hodosy, Roland Pálffy, Roman Gardlík, Lukáč Halčák, Daniela Ostatníková

**Affiliations:** 1 Institute of Molecular Biomedicine, Comenius University, Bratislava 811 08, Slovakia; E-Mails: hodosy@gmail.com (J.H.); palffyroli@gmail.com (R.P.); romangardlik@gmail.com (R.G.); 2 Institute of Pathophysiology, Comenius University, Bratislava 811 08, Slovakia; 3 Department of Molecular Biology, Comenius University, Bratislava 811 08, Slovakia; 4 Institute of Physiology, Comenius University, Bratislava 811 08, Slovakia; E-Mail: daniela.ostatnikova@fmed.uniba.sk; 5 Institute of Chemistry, Biochemistry and Clinical Biochemistry, Comenius University, Bratislava 811 08, Slovakia; E-Mail: lukac.halcak@fmed.uniba.sk

**Keywords:** soy, phytoestrogens, AOPP, AGEs, antioxidative status

## Abstract

Beyond other beneficial effects, a soy-rich diet has been shown to reduce the risk of cardiovascular diseases and diabetic complications. Reduction of oxidative and carbonyl stress has been proposed as the underlying mechanism, but the evidence for this is lacking. The aim of our study was to evaluate the effects of short-term increased soy intake on oxidative and carbonyl stress parameters in young volunteers. Young healthy probands (omnivores) of both genders (55 women, 33 men) were given soybeans (2 g/kg bodyweight daily) for one week. Markers of oxidative and carbonyl stress were measured in plasma at the beginning and at the end of one week soybean intake and after another week of a wash-out period. Total antioxidant capacity was increased by soybean intake in both genders. This led to decreased levels of advanced oxidation protein products in women, but not in men. On the contrary, in men, soybean intake increased lipoperoxidation. No effects on carbonyl stress markers (advanced glycation end products-specific fluorescence and fructosamine) were found. Soybean intake has gender-specific effects on oxidative stress in young healthy probands potentially due to divergent action and metabolism of phytoestrogens in men and women. Effects of soybean intake on carbonyl stress should be evaluated in longer studies.

## 1. Introduction

Soy-rich diet has well documented effects on menopausal symptoms [1,2], osteoporosis [3,4] and breast cancer [5,6]. Soy phytoestrogens have been previously shown to reduce cardiovascular risk by decreasing cholesterol levels [7,8], blood pressure [9] and improving endothelial function [10,11]. Increased soy intake has even been proposed as prevention of cardiovascular diseases [12,13].

One of the potential mechanisms mediating these effects might be reduced oxidative stress [14,15]. Numerous animal experiments showed protective effects of soy constituents on oxidative stress [16,17]. Results from clinical studies are not that clear. Most of the published data report beneficial effects [18,19], but in some studies, however, no significant effects were found [20,21]. Trials targeted on the effects of soy phytoestrogens on oxidative stress were performed mostly on postmenopausal women [22,23,24]. In populations where soy intake is proposed as prevention of breast cancer—premenopausal women [25], or prostate cancer—men [26,27], most of the studies report small numbers of participants [21,28]. None of the studies focused on the short-term effects of increased soy intake.

There are some data indicating an antidiabetic effect of soy phytoestrogens, both in an animal model [29] and in a human epidemiological study [30]. However, studies focusing on carbonyl stress and advanced glycation as a crucial pathogenic mechanism in diabetic patients are lacking. Carbonyl stress, beyond other pathways, can be induced by oxidative stress as some products of oxidative damage contain carbonyl moieties which can substitute glucose in the Maillard reaction. We, thus, hypothesized that soy intake may reduce both, oxidative and carbonyl stress. To characterize oxidative stress we measured the levels of thiobarbituric reactive substances (TBARS) as a marker of lipid peroxidation, advanced oxidation protein products (AOPP) as marker of protein oxidation and total antioxidant capacity (TAC) as a measure of the antioxidant status. TBARS and AOPP describe the level of oxidative damage to lipids and proteins, respectively. TAC is the most widely used marker of the ability of plasma to resist oxidative damage and is affected by the concentration of both, low and high molecular weight antioxidants. Advanced glycation end products (AGEs) are end products of the Maillard reaction between amino-groups of macromolecules and free carbonyl compounds. Fructosamine is one of the Maillard reaction products. The aim of our study was to evaluate the effects of short-term soy intake on oxidative and carbonyl stress parameters in young healthy men and women.

## 2. Results and Discussion

**Figure 1:**
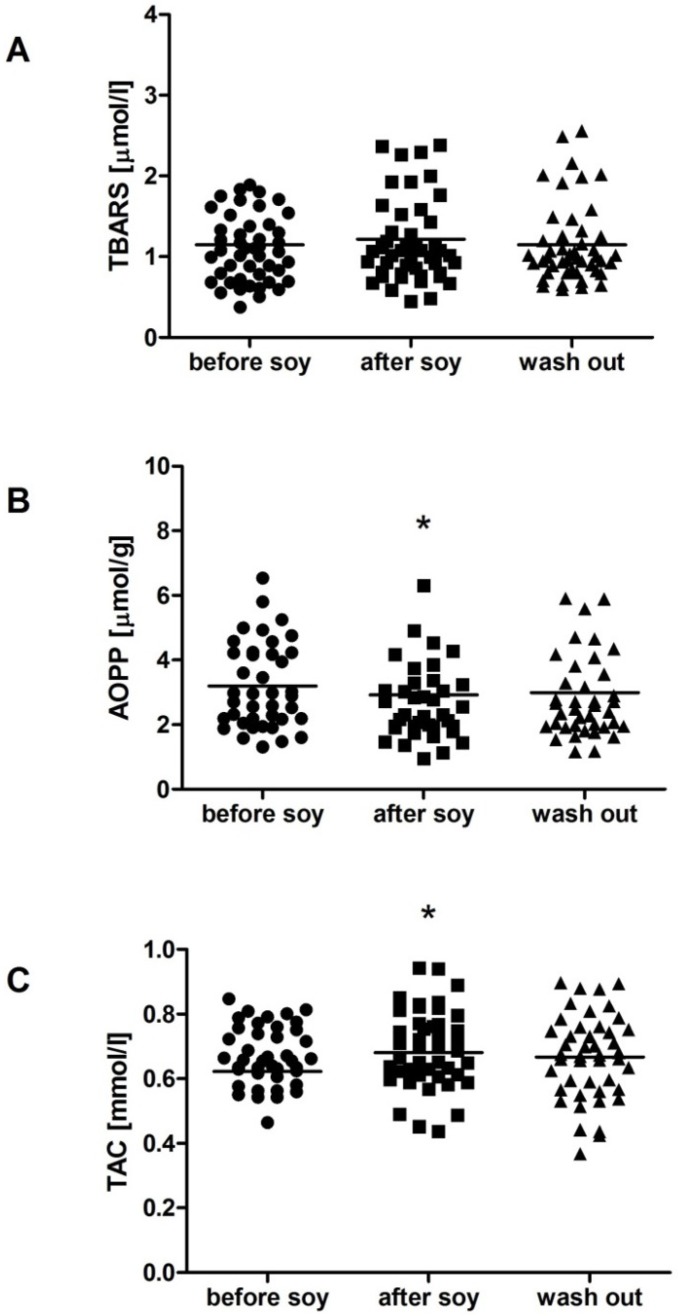
Oxidative stress parameters in women. Soy intake did not affect thiobarbituric acid reactive substances (TBARS) levels (**A**), but reduced advanced oxidation protein products (AOPP), while the effect persisted one week after soybean intake (**B**). Total antioxidant capacity of plasma (TAC) was increased after one week of soybean intake (**C**). * denotes *p* < 0.05 *vs.* before soy.

**Figure 2:**
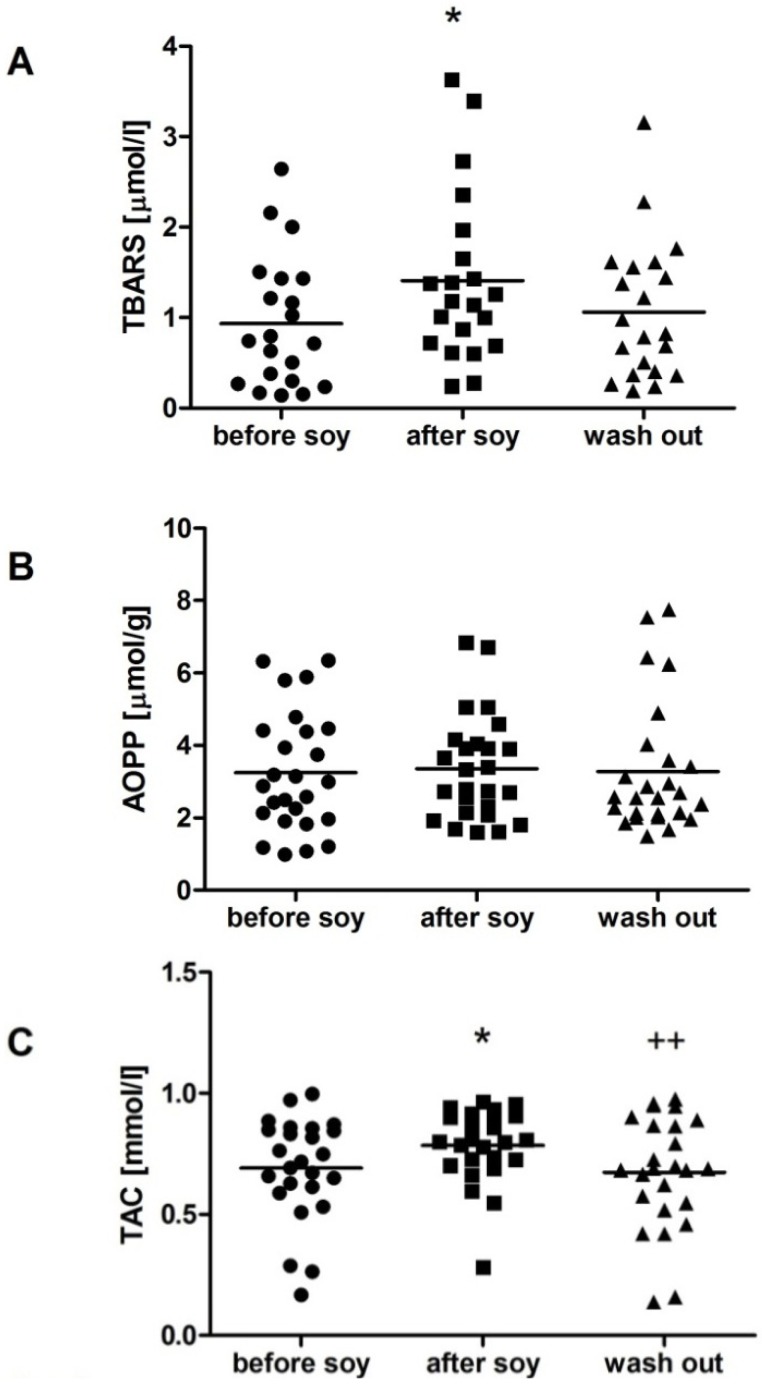
Oxidative stress parameters in men. Soy intake increased thiobarbituric acid reactive substances (TBARS) levels (**A**), but had no effect on advanced oxidation protein products (AOPP; **B**). Total antioxidant capacity of plasma (TAC) was increased by one week soybean intake (**C**). * denotes *p* < 0.05 *vs.* before soy; ^++^ denotes *p* < 0.01 *vs.* after soy.

**Figure 3:**
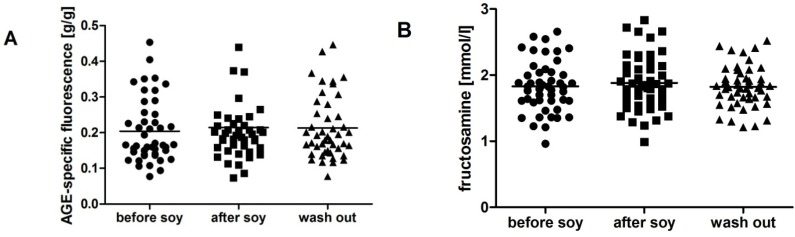
Carbonyl stress parameters in women. Soy intake did not affect advanced glycation end products (AGE-specific fluorescence; **A**). No changes were found in plasma fructosamine levels (**B**).

**Figure 4:**
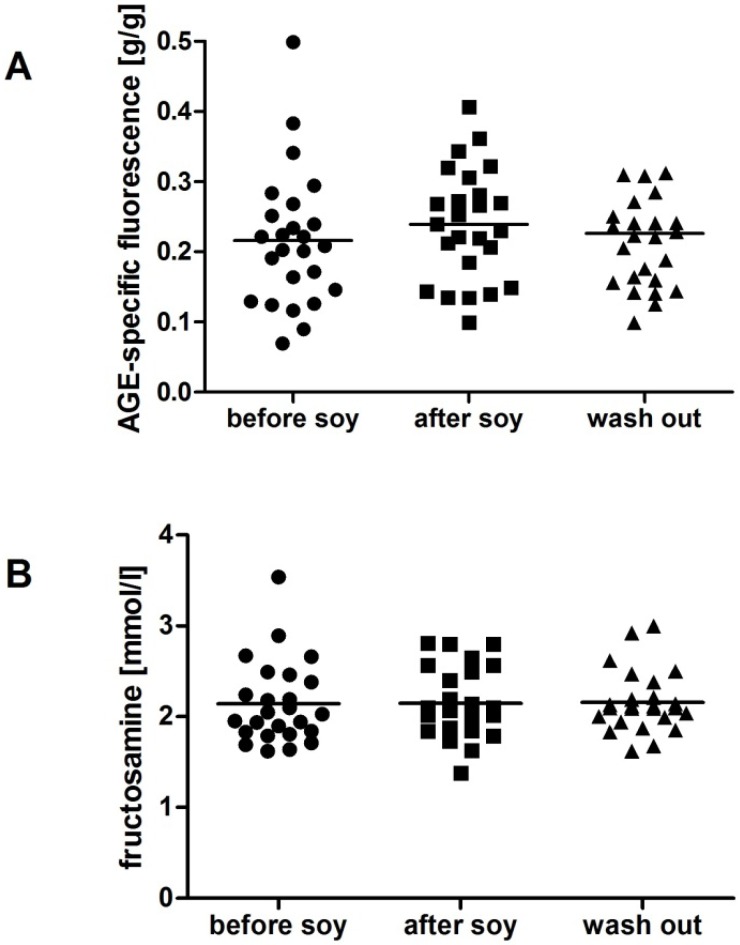
Carbonyl stress parameters in men. Soy intake did not affect advanced glycation end products (AGE-specific fluorescence; **A**). No changes were found in plasma fructosamine levels (**B**).

We have found no gender differences in the analyzed parameters at the beginning of the study except for fructosamine, which was higher in men during the whole study (*p* < 0.05). TBARS as a marker of lipid peroxidation was not affected by soybean intake in women (Figure 1A), but increased in men by 50% after one week soybean intake, returning to nearly baseline values after the wash-out period (Figure 2A; F = 3.5; *p* < 0.05). AOPP as a marker of oxidative damage of proteins was decreased by soybean intake in women by 12%. The decrease persisted after the wash-out period, although the difference was marginally non-significant (Figure 1B; F = 3.9; *p* = 0.09). In contrast, no significant dynamics was detected in AOPP in men (Figure 2B). In both genders TAC was increased significantly by soybean intake. In women the increase was approximately 10% and persisted after the wash-out period (Figure 1C; F = 3.6; *p* < 0.05). In men TAC increased by 13%, but decreased during the wash-out period to baseline values (Figure 2C; F = 6.1; *p* < 0.01). No significant changes were found in carbonyl stress markers AGE-specific fluorescence and fructosamine, neither in women (Figure 3), nor in men (Figure 4). 

The results of this study indicate that one week of high soybean intake increases antioxidative status in men and women as measured by TAC in plasma. Although the increase is relatively small, it is in line with previous studies focusing on nutritional effects on antioxidative status in healthy subjects [31]. Given the short duration of the intervention, this small increase might have physiological consequences, although these were not assessed in our study. The physiological relevance of this result can hardly be evaluated without further functional assessments, especially as methods for measuring the antioxidative status are not quantitatively harmonized. The extrapolation of the functional effects of small changes in antioxidative status in other studies is not possible due to differences in measurements and quantification of the antioxidative status. In line with the changes of antioxidative status is the reduction of AOPP in women. On the contrary, in men, AOPP was not changed and TBARS were even increased by soybean intake. Gender-specific effects of soybean intake might be explained by the different hormonal milieu in young men and women. Phytoestrogens are known to bind to estrogen receptors. In low estrogen environment (men or postmenopausal women) phytoestrogens can act as estrogen receptor agonists. In high estrogen environment (premenopausal women) the weak binding functionally results in estrogen antagonism [32]. Estradiol has been shown to act as antioxidant or prooxidant depending on the physiological context [33]. The subtle effects of soybean intake on endocrine parameters were published by our group previously [34]. However, looking for gender differences was not the primary aim of our study, so the design with a different number of participating men and women might bias our gender-specific results.

Previous studies focusing on the effects of soy have shown that soy intake decreases oxidative stress. In a double-blind randomized study on postmenopausal women no effects of soy protein or its constituents on oxidative stress parameters were seen [23]. In our study, we found no effect of soybean intake on TBARS levels in women. On the other hand, soy decreased AOPP in women, but not in men. AOPP are mostly the products of protein oxidation by hypochlorous acid [35]. Interestingly, estradiol was reported to induce myeloperoxidase—the major source of hypochlorous acid in neutrophils [36]. Although this is only a speculation, as neither hypochlorous acid nor neutrophil activity was measured, it might point towards the potential mechanism behind the observed effects of soybean intake on AOPP in women. Animal experiments and *in vitro* analysis should prove whether this potential explanation is correct.

Gender specific effect of soybean intake on AOPP might be explained also by the divergent effects of soy phytoestrogens in men and women. Phytoestrogens are metabolized by the gut microflora that varies between individuals [37]. Gender differences in the microbiome could cause differences in phytoestrogen metabolism that led to a gender-specific effect of soybean intake on oxidative stress markers [38]. This might also be the reason for the lack of effects of soybean intake on TBARS in women as well as on AOPP in men. The phytoestrogen levels in our study were increased by soybean intake as reported elsewhere [39]. Unfortunately, specific phytoestrogen metabolites were not measured in this study.

Only a few studies have been published on the effects of soy in men. In a small 4 week long study soy intake protected DNA from oxidative damage in healthy men [19]. This has been proposed as the mechanism behind cancer preventive effects of soy [18]. However, these effects are still not fully understood [27]. Acute postprandial oxidative stress was not reduced by soy intake in men with hypertriglyceridemia [20]. In line with these results the levels of AOPP in men were not reduced in our study. This fact might be the consequence of the short duration of our study. In addition, despite increased TAC, the TBARS levels were increased by soybean intake. The potential genotoxic effects of phytoestrogens and especially some of the phytoestrogen metabolites have been described in the past *in vitro* [40]. Depending on the microbial metabolism in the gut and on the activity of the hepatic microsomes the oxidative metabolites might be formed that are able to attack nucleic acids and phospholipids. Especially, genistein and some of its derivatives were shown to be able to induce genetic mutations [41]. This might point towards the mechanism behind the observed increased TBARS levels in men.

According to our knowledge this is the first study to analyze the effects of soybean intake on parameters of carbonyl stress. We have found no effect on AGEs or fructosamine in either gender. However, the soybean intake lasted only one week and the participants were young and healthy. We are aware of this limitation. The lack of effects of soybean intake on carbonyl stress might be a consequence of the short duration of the study. A longer study might be needed as the dynamics of the products of non-enzymatic glycation is usually slow. Future longer studies, potentially focusing on metabolic disorders such as diabetes might be more sensitive in this aspect. Analyses of additional and more specific markers of oxidative and carbonyl stress might increase the sensitivity of the study. Beyond short duration of the study one of the weaknesses is that the food habits of the participants were not monitored in detail. On the other hand, we did not want to introduce bias by artificially changing other nutritional factors, although very probably some of the effects seen might be caused by the omission of some food by the participants during the intervention.

## 3. Experimental

Young healthy women (n = 55) and men (n = 33) were recruited among university students. All participants were between 18 and 25 years of age. Exclusion criteria were vegetarianism, any known pathologies, medications, hormonal contraception and regular intake of food supplements including vitamins. The participants were given commercially available soybeans (AlfaBio, Bratislava, Slovakia) and were asked to eat 2 grams (dry weight) of soybeans per kilogram bodyweight daily during 7 consecutive days without any restriction regarding preparation or other food intake. The dose was chosen based on a preliminary study and preferences of volunteers. Peripheral venous blood samples were taken before soybean intake, after one week of soybean intake and after a wash-out period of another 7 days. All samplings were performed between 8:00 and 9:00 to prevent any bias from circadian dynamics. Blood samples were centrifuged (5,000 g, 5 min), plasma was aliquoted into tubes and stored frozen until analysis. All participants signed a written informed consent. The study protocol was approved by the local Ethics committee of the Institute of Molecular Biomedicine, Comenius University.

### 3.1. Oxidative Stress Markers

TBARS were measured spectrophotometrically. Plasma samples were incubated with thiobarbituric acid (TBA) in a mixture with acetic acid for 45 minutes at 95 °C [31]. Malondialdehyde-TBA adducts were recovered with *n*-butanol and quantified at 532 nm against a calibration curve (1,1,3,3-tetramethoxypropane). 

AOPP were quantified at 340 nm after 2 min incubation of samples with acetic acid [32]. Calibration curve was prepared from absorbances of chloramine T mixtures with potassium iodide. Data were normalized to protein levels. 

TAC was determined in a coloured reaction between (2,2'-azino-bis(3-ethylbenzthiazoline-6-sulphonic acid) ABTS and hydrogen peroxide in acetate buffer [33]. After 5 min the absorbance was taken at 660 nm against Trolox calibration curve. 

### 3.2. Carbonyl Stress Markers

AGEs were quantified by spectrofluorometry [34]. AGE-specific fluorescence was determined in samples diluted in phosphate buffered saline at 370 nm (excitation) and 440 nm (emission). AGE modified bovine serum albumin (AGE-BSA) was used as a standard. Data were normalized to protein levels. 

Fructosamine was measured in samples incubated with nitro blue tetrazolium in sodium carbonate buffer for 15 min at 37 °C [35]. The concentrations were calculated from absorbance values at 530 nm against the fructosamine (1-deoxymorpholino-d-fructose) standard.

### 3.3. Statistical Analysis

Data are presented as Mean + Standard Error of the Mean. Analysis of the dynamics was performed using repeated measures ANOVA and *post hoc* Tukey test for comparison of time points. P-values less than 0.05 were considered significant. All calculations were done using the GraphPad Prism 5.02 software.

## 4. Conclusions

In conclusion, our study showed that one week soybean intake increases TAC in both genders and lowers protein oxidation in women, but not in men. In addition, soybean intake increased lipid peroxidation in men. Whether these changes have any long-term consequences is currently unknown. The finding that soy intake has no effects on carbonyl stress must be taken with caution due to the short duration of soybean intake in our study. Further longer studies focusing especially on carbonyl stress are needed.
